# Nanoarchitectonics and Biological Properties of Nanocomposite Thermosensitive Chitosan Hydrogels Obtained with the Use of Uridine 5′-Monophosphate Disodium Salt

**DOI:** 10.3390/ijms25115989

**Published:** 2024-05-30

**Authors:** Katarzyna Pieklarz, Grzegorz Galita, Ireneusz Majsterek, Piotr Owczarz, Zofia Modrzejewska

**Affiliations:** 1 Department of Environmental Engineering, Faculty of Process and Environmental Engineering, Lodz University of Technology, 93-005 Lodz, Poland; 2 Department of Clinical Chemistry and Biochemistry, Medical University of Lodz, 92-215 Lodz, Poland; grzegorz.galita@umed.lodz.pl (G.G.); ireneusz.majsterek@umed.lodz.pl (I.M.); 3 Department of Chemical Engineering, Faculty of Process and Environmental Engineering, Lodz University of Technology, 93-005 Lodz, Poland; piotr.owczarz@p.lodz.pl

**Keywords:** tissue engineering, thermosensitive hydrogel, rheology, structural properties, biocompatibility, chitosan, uridine 5′-monophosphate disodium salt, graphene oxide

## Abstract

Currently, an important group of biomaterials used in the research in the field of tissue engineering is thermosensitive chitosan hydrogels. Their main advantage is the possibility of introducing their precursors (sols) into the implantation site using a minimally invasive method—by injection. In this publication, the results of studies on the new chitosan structures in the form of thermosensitive hydrogels containing graphene oxide as a nanofiller are presented. These systems were prepared from chitosan lactate and chitosan chloride solutions with the use of a salt of pyrimidine nucleotide—uridine 5′-monophosphate disodium salt—as the cross-linking agent. In order to perform the characterization of the developed hydrogels, the sol–gel transition temperature of the colloidal systems was first determined based on rheological measurements. The hydrogels were also analyzed using FTIR spectroscopy and SEM. Biological studies assessed the cytotoxicity (resazurin assay) and genotoxicity (alkaline version of the comet assay) of the nanocomposite chitosan hydrogels against normal human BJ fibroblasts. The conducted research allowed us to conclude that the developed hydrogels containing graphene oxide are an attractive material for potential use as scaffolds for the regeneration of damaged tissues.

## 1. Introduction

In recent years, in addition to interest in the application possibilities of chitosan (CH) in medical sciences, there has been an increase in research undertaken in the field of nanomedicine. Of particular interest are chitosan composites containing nanostructured carbon materials, mainly graphene and graphene oxide (GO).

Graphene, an allotropic form of carbon, occurs in the form of a two-dimensional carbon monolayer with a thickness of 0.334 nm. Its honeycomb-like structure results from covalent bonds between carbon atoms with electron orbitals that occur in sp^2^ hybridization [1,2]. It is characterized by unique properties, such as a high specific surface area (~2600 m^2^·g^−1^), high electron mobility (200,000 cm^2^·V^−1^·s^−1^), high thermal conductivity (3000–5000 W·m^−1^·K^−1^), and exceptional mechanical strength (a Young’s modulus of 1 TPa) [2]. However, one should be aware that the main obstacle to the use of graphene as a component of medical products intended for contact with living organisms is its hydrophobic nature [1,3]. Graphene in body fluids changes its spatial conformation and undergoes precipitation. Therefore, GO is increasingly used, which, unlike graphene, has a hydrophilic nature [3].

GO is an oxidized, layered carbon structure with the characteristic hydroxyl, carboxyl, epoxy, and carbonyl functional groups [1,4], thanks to which it is characterized by high dispersibility in the aqueous environment and can interact with many polymers, and additionally, like graphene, it exhibits unusual physical, chemical, and mechanical properties [1,5]. Its biological properties should also be emphasized, such as its antibacterial [6,7] and anticancer [8,9] activities. For this reason, there is great potential for the use of GO in medicine and related fields, including as a component of antibacterial materials, a carrier for drug and gene delivery, in tissue engineering, in biosensors, or for the selective imaging of specific tissue areas [10,11,12,13,14].

Nevertheless, the issue of the biocompatibility and toxicity of graphene materials, including GO, is still controversial. Some researchers indicate that GO is biocompatible, while others report its cytotoxicity to cells from various tissues and organs [15,16]. However, it is common knowledge that graphene materials should be biocompatible with healthy tissues, while anticancer and antibacterial therapies require the use of materials with high toxicities to cancer and bacterial cells. Therefore, there is a need to elucidate the mechanisms responsible for toxicity [1].

Despite the many discrepancies related to the biocompatibility and toxicity of GO, much hope is placed in its use as a nanofiller of chitosan systems, which, due to the high biocompatibility of the polymer [10,17], would eliminate the potential toxicity of GO [18]. Due to the presence of oxygen functional groups on the GO surface, it can, through covalent bonds, hydrogen bonds, or electrostatic interactions, create composites with CH, which reduce the limitations of both components (the low mechanical strength of the polymer and the potentially low biocompatibility of GO). Through synergistic interactions between CH and GO, systems with high thermal stability, satisfactory mechanical and optical properties [19,20], excellent biocompatibility [21,22], and good antibacterial properties [23] are created.

In this publication, nanocomposite thermosensitive chitosan hydrogels containing GO as a nanofiller are characterized. These systems were prepared from chitosan lactate and chitosan chloride solutions with the use of uridine 5′-monophosphate disodium salt (UMP) as a cross-linking agent.

First, rheological measurements were performed to determine the sol–gel transition points of the prepared chitosan systems. Moreover, the hydrogels were characterized by Fourier transform infrared spectroscopy (FTIR) and scanning electron microscopy (SEM). In turn, the in vitro biological tests included the assessment of the cytotoxic and genotoxic effects of the developed biomaterials against normal human BJ fibroblasts.

The presented research aimed to check whether the introduction of GO into the polymer matrix improves the mechanical and structural properties and affects the biocompatibility of the modified systems compared to hydrogels without GO.

## 2. Results and Discussion

### 2.1. Rheological Measurements of the Colloidal Chitosan Salt Solutions Containing GO

**Figure 1:**
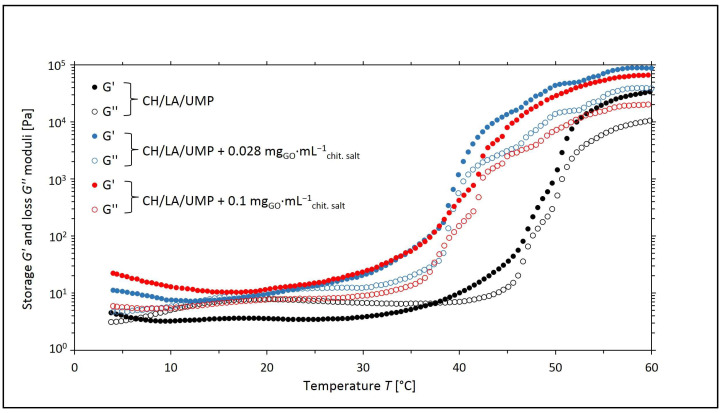
The experimental curves of the changes in the values of the storage (*G*′) and loss (*G*″) moduli as a function of temperature (*T*) obtained for chitosan lactate solutions (the CH/LA/UMP systems) containing different concentrations of GO.

**Figure 2:**
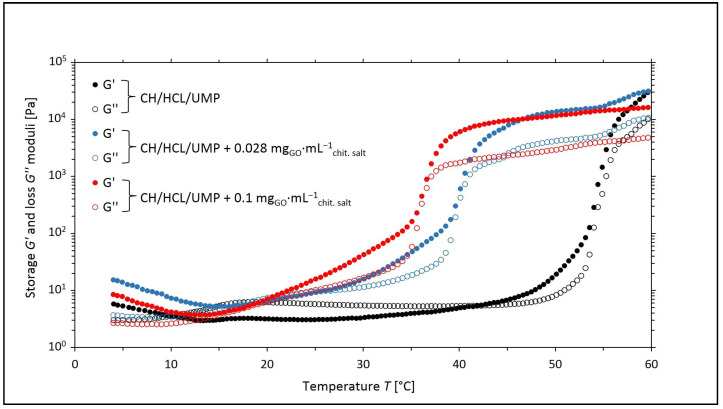
The experimental curves of the changes in the values of the storage (*G*′) and loss (*G*″) moduli as a function of temperature (*T*) obtained for chitosan chloride solutions (the CH/HCL/UMP systems) containing different concentrations of GO.

Figure 1 and Figure 2 show the changes in the values of the storage (*G*′) and loss (*G*″) moduli as a function of temperature (*T*) for the systems with UMP containing GO with mass concentrations of 0.028 and 0.1 mg_GO_·mL^−1^_chit. salt_, respectively.

From the course of the curves of *G*′ = *f*(*T*) and *G*″ = *f*(*T*) for the CH/LA/UMP and CH/HCL/UMP systems containing 0.028 mg_GO_·mL^−1^_chit. salt_, four regions are indicated, similar to the unmodified systems discussed in our publications [24,25], that characterize the processes that occur in the samples during the measurement:

▪ Region 1—*G*′ moduli dominate over *G*″ moduli, and the systems exhibit typical gel behavior: flexible networks are formed;
▪ Region 2 (the sol area)—the viscous properties prevail over the elastic ones;
▪ Region 3—further increasing the temperature leads to the initiation of the sol–gel transition process (*G*′ > *G*″);
▪ Region 4—the gelation process occurs more slowly.

In turn, for the CH/LA/UMP and CH/HCL/UMP systems containing 0.1 mg_GO_·mL^−1^_chit. salt_, over the whole temperature range, the elastic properties (*G*′ moduli) prevail over the viscous ones (*G*″ moduli). For this reason, the characteristic regions of the sol–gel transition cannot be distinguished.

**Figure 3:**
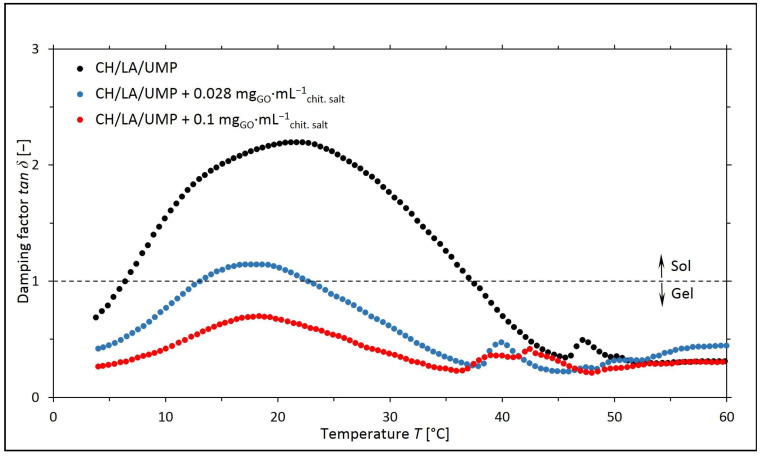
The experimental curves of the changes in the values of the damping factor (*tan δ* = *G*″/*G*′) as a function of temperature (*T*) obtained for chitosan lactate solutions (the CH/LA/UMP systems) containing different concentrations of GO.

**Figure 4:**
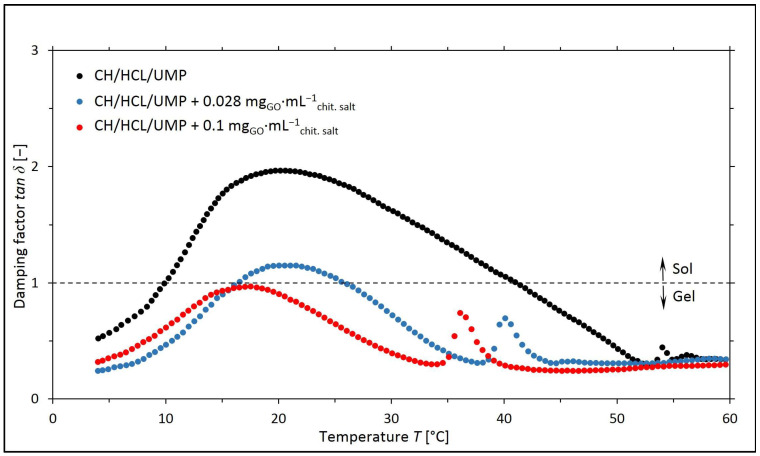
The experimental curves of the changes in the values of the damping factor (*tan δ* = *G*″/*G*′) as a function of temperature (*T*) obtained for chitosan chloride solutions (the CH/HCL/UMP systems) containing different concentrations of GO.

Based on the values of the *G*′ and *G*″ moduli, the corresponding values of the damping factor (*tan δ* = *G*″/*G*′) for a given temperature were determined, and the curves of the *tan δ* = *f*(*T*) are illustrated in Figure 3 and Figure 4.

The obtained research results indicate that for the chitosan salt solutions (lactate and chloride) containing 0.028 mg_GO_·mL^−1^_chit. salt_, the sol–gel transitions occurred at lower temperatures than for the systems without nanofiller. However, in the case of the samples containing a higher concentration of GO, over the whole temperature range, *tan δ* < 1, and therefore the elastic properties dominate over the viscous ones.

The gelation temperature values obtained for the chitosan systems are listed in Table 1.

The results of the rheological measurements carried out for the colloidal chitosan salt solutions containing UMP indicate that the addition of GO to the solutions reduces the sol–gel transition temperature while improving the mechanical properties of the hydrogels (the storage (*G*′) moduli reach a higher value than in the case of systems without nanofiller). This is a reason to assume that the introduction of GO into the chitosan salt systems may prove beneficial during the potential use of the above solutions as precursors of injection scaffolds formed in vivo at the site of tissue damage [25].

### 2.2. Structural Characteristics of the Modified Chitosan Hydrogels

**Figure 5:**
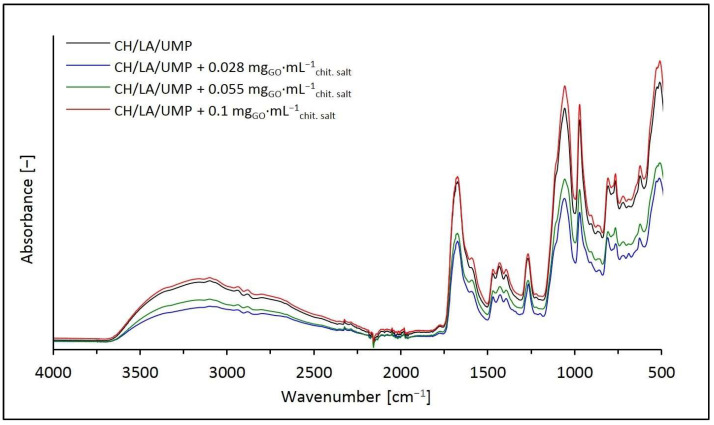
FTIR spectra of the CH/LA/UMP systems containing different concentrations of GO in the wavenumber range of 4000–500 cm^−1^.

The FTIR spectra of the lyophilized hydrogels obtained from the chitosan lactate solutions with UMP containing the addition of GO and, as a reference point, the spectrum of the unmodified system (the CH/LA/UMP system) are illustrated in Figure 5.

Analyzing the above spectra, it can be noticed that the introduction of GO into the chitosan lactate solutions only contributed to differences in the intensity of the absorption signals identified for the control sample (the CH/LA/UMP system), discussed in detail in our publications [25,26,27]. This proves the good dispersion of the nanofiller into the polymer matrix.

In the spectra of hydrogels containing GO with mass concentrations of 0.028 and 0.055 mg_GO_·mL^−1^_chit. salt_, the intensities of the bands occurring in the wavenumber range of 3600–3000 cm^−1^ (the broad, asymmetric band corresponding to the O–H stretching) and in the region of 2950–2850 cm^−1^ (the region bound with symmetric and asymmetric stretching vibrations of the –CH groups from the chitosan pyranose ring and stretching vibrations of the C–H bonds in the –CH_2_– and –CH_3_ groups assigned to the ring) are lower than those for the CH/LA/UMP system, and for the sample containing 0.1 mg_GO_·mL^−1^_chit. salt_, the intensity is slightly higher.

Similarly, to the above areas, the intensities of the absorption bands occurring in the wavenumber range of 1750–500 cm^−1^ (the range characteristic of UMP) for the samples containing 0.028 and 0.055 mg_GO_·mL^−1^_chit. salt_ are also lower than those for the unmodified sample. However, for a concentration of 0.1 mg_GO_·mL^−1^_chit. salt_, which is particularly noticeable at wavenumbers such as 1050 cm^−1^ with an arm at 1100 cm^−1^ (stretching vibrations of the C(4′)–C(5′)–O moiety, and the signal of 1100 cm^−1^ may additionally indicate the presence of the –PO_2_^–^ group), 970 cm^−1^ (symmetric stretching vibrations of the –PO_3_^2–^ group), or 510 cm^−1^ (bending vibrations of the uracil ring and uracil carbonyl groups), a clear increase is visible in the intensity compared to the signals identified for the CH/LA/UMP system without nanofiller.

**Figure 6:**
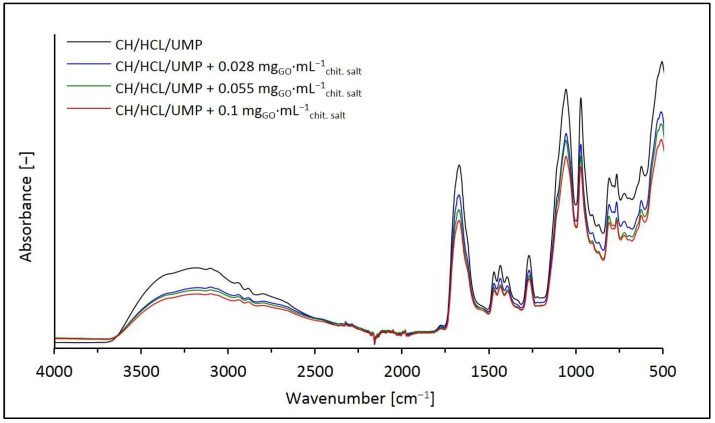
FTIR spectra of the CH/HCL/UMP systems containing different concentrations of GO in the wavenumber range of 4000–500 cm^−1^.

Figure 6 shows the FTIR spectra of the hydrogels obtained from the chitosan chloride solutions with UMP containing GO, and the spectrum of the sample without nanofiller (the CH/HCL/UMP system) as a reference point.

Considering the above spectra, it can be concluded that the introduction of GO into the chitosan chloride solutions with UMP, similar to the previously discussed hydrogel variants, only contributed to changes in the intensity of the absorption signals identified for the control sample (the CH/HCL/UMP system). As the GO concentration increased, the intensities of the bands occurring in the wavenumber ranges of 3600–3000 cm^−1^ and 2950–2850 cm^−1^ and in the region of 1750–500 cm^−1^ decreased.

### 2.3. Morphological Characteristics of the Chitosan Hydrogels Containing GO

**Figure 7:**
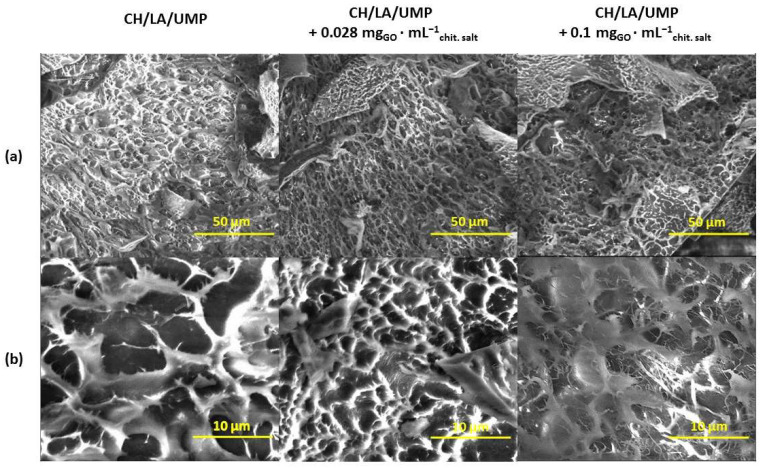
SEM micrographs of the CH/LA/UMP system without and with nanofiller (magnification: (**a**) 1000×, (**b**) 5000×).

**Figure 8:**
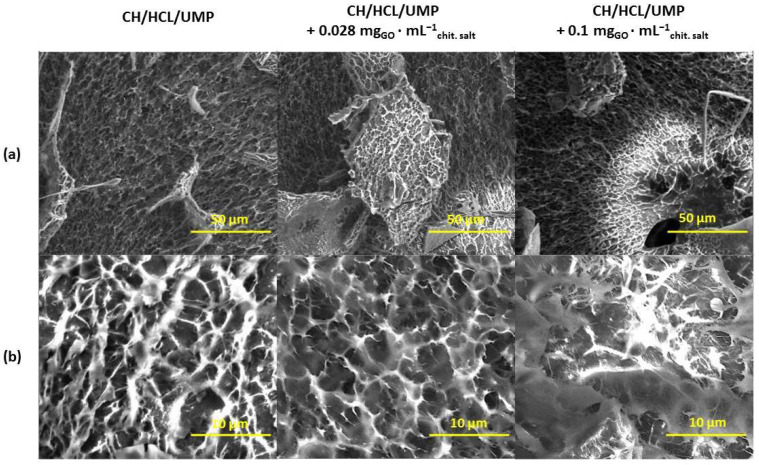
SEM micrographs of the CH/HCL/UMP system without and with nanofiller (magnification: (**a**) 1000×, (**b**) 5000×).

Figure 7 shows micrographs of the morphologies of the hydrogels prepared from the chitosan lactate solutions using UMP, containing 0.028 and 0.1 mg_GO_·mL^−1^_chit. salt_, and, as a reference point, photos of the system without nanofiller. In turn, Figure 8 shows microimages of the modified samples obtained from the chitosan chloride with UMP.

Based on the above images, it can be concluded that the structure of all modified hydrogels, regardless of the type of solvent or nanofiller concentration, is like the structure of samples without GO. The scaffolds exhibit a uniform, interconnected architecture with the pores evenly distributed over the entire surface. In the case of the systems prepared from chitosan lactate and chitosan chloride solutions containing 0.1 mg_GO_·mL^−1^_chit. salt_ (Figure 7b and Figure 8b), the presence of fibrous elements can also be observed in their structure. 

These observations support the assumption that chitosan systems with UMP are an interesting material for potential use in biomedical engineering. One of the key parameters of scaffolds is their architecture, including the existence of pores and their arrangement. The presence of open porosity in scaffolds allows for cell migration and growth, ensures the proper vascularization of the newly formed tissue and the effective delivery of nutrients, and enables the diffusion of gases and the removal of metabolic products [28].

### 2.4. Evaluation of the In Vitro Biocompatibility of Hydrogels

In vitro biocompatibility tests are a key element of the research on implant materials and are also the first preclinical stage related to the development of new solutions with commercialization potential. This is due to the fact that biomaterials, in addition to the appropriate structural properties, must first of all demonstrate the ability to coexist with the body’s natural tissues, providing an environment with features similar to those that characterize the recipient’s own tissue [1,25]. 

**Figure 9:**
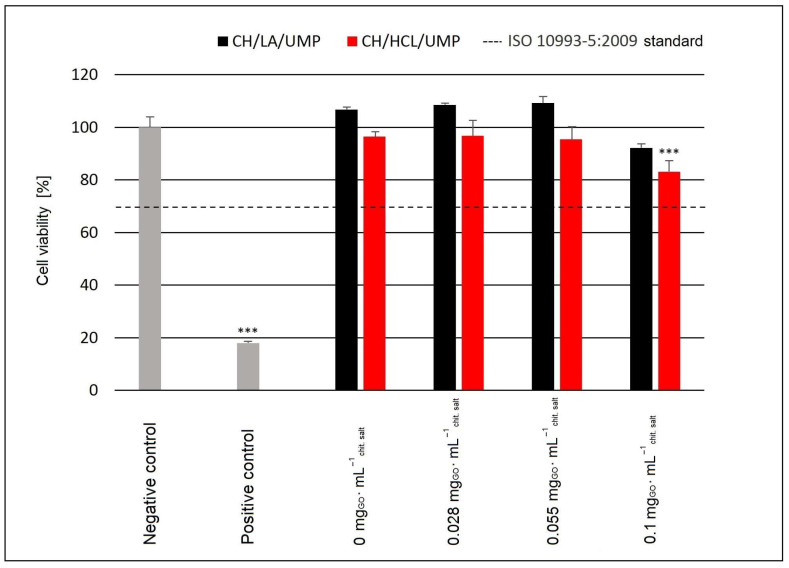
The viability of BJ cells after 48 h of incubation with the chitosan hydrogels with UMP containing different concentrations of GO; *** *p* < 0.001 versus negative control.

As part of the biological studies, the cytotoxic and genotoxic effects of the developed chitosan hydrogels against normal human BJ fibroblasts were evaluated. The results of the cytotoxicity tests are shown in Figure 9.

The cytotoxicity analysis, measured by a test based on the reduction of resazurin to resorufin [27], showed that after 48 h of the incubation of BJ cells with the samples of the CH/LA/UMP system containing the GO concentrations 0 (sample without nanofiller), 0.028, 0.055, and 0.1 mg_GO_·mL^−1^_chit. salt_, cell viability at the levels of 106.80 ± 0.88%, 108.54 ± 0.62%, 109.22 ± 2.47%, and 92.24 ± 1.47%, respectively, was achieved. No statistically significant differences between the fibroblast’s viability exposed to the above biomaterials and the negative control were noted. All systems prepared from the chitosan lactate solutions with UMP, regardless of the GO concentration, did not induce cytotoxic effects on the BJ cells, meeting both the provisions of the ISO 10993-5:2009 standard [29] and the more restrictive criterion given by Dahl et al. [30]. It should be emphasized that low concentrations of nanofiller (0.028 and 0.055 mg_GO_·mL^−1^_chit. salt_) in the case of the CH/LA/UMP system promote the survival of BJ cells at a level above 100%. 

In contrast, the hydrogels obtained from the chitosan chloride solutions containing the highest concentration of GO showed low cytotoxicity. For these systems, the cell viability was 83.09 ± 4.22%, which turned out to be statistically significant in relation to the negative control (*** *p* < 0.001). In the case of the remaining variants of the CH/HCL/UMP system, the fibroblast survival exceeded 92% (no signs of cytotoxicity), which was not statistically significant compared to the negative control.

Due to the fact that the analyzed hydrogels with UMP containing a carbon nanofiller constitute a modern group of materials that use solutions offered by nanotechnology, genotoxicity tests using the alkaline version of the comet assay were performed [27]. 

**Figure 10:**
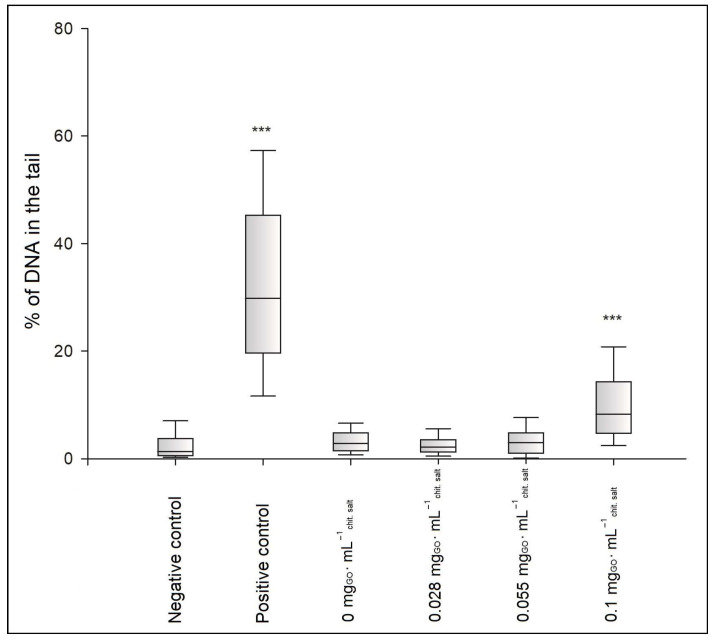
Degree of DNA damage in BJ cells after 48 h of incubation with the hydrogels of the CH/LA/UMP system containing different concentrations of GO; *** *p* < 0.001 versus negative control.

**Figure 11:**
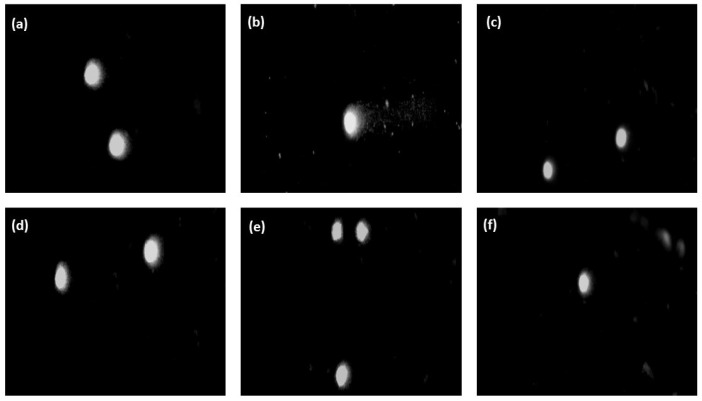
Representative images of comets (200× magnification) obtained in the alkaline version of the comet assay after 48 h of incubation of BJ cells with the hydrogels of the CH/LA/UMP system containing various concentrations of GO: (**a**) negative control; (**b**) positive control; (**c**) 0 mg_GO_·mL^−1^_chit. salt_ (sample without nanofiller); (**d**) 0.028 mg_GO_·mL^−1^_chit. salt_; (**e**) 0.055 mg_GO_·mL^−1^_chit. salt_; (**f**) 0.1 mg_GO_·mL^−1^_chit. salt_.

**Figure 12:**
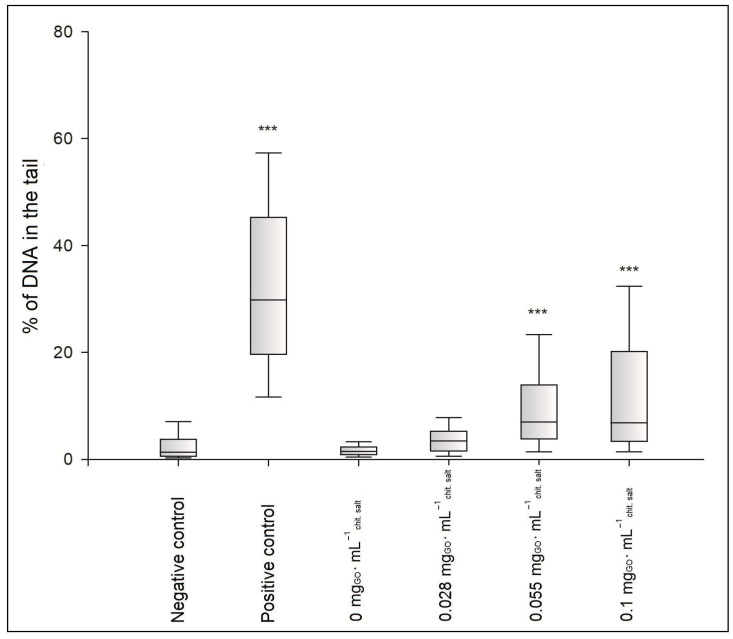
Degree of DNA damage in BJ cells after 48 h of incubation with the hydrogels of the CH/HCL/UMP system containing different concentrations of GO; *** *p* < 0.001 versus negative control.

**Figure 13:**
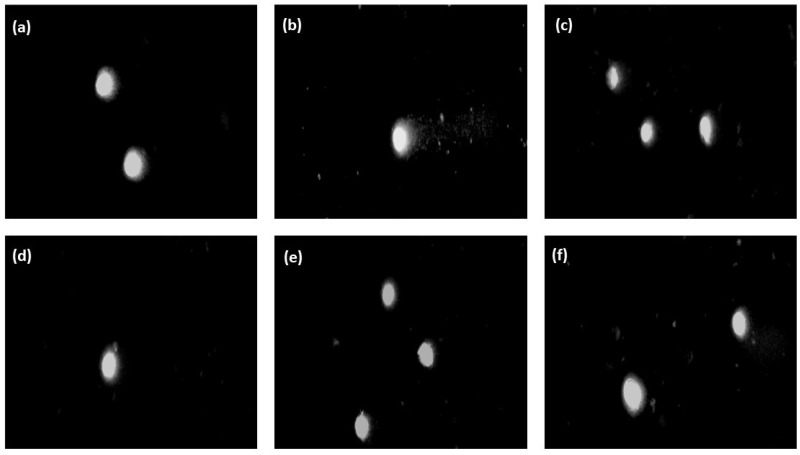
Representative images of comets (200x magnification) obtained in the alkaline version of the comet assay after 48 h of incubation of BJ cells with the hydrogels of the CH/HCL/UMP system containing various concentrations of GO: (**a**) negative control; (**b**) positive control; (**c**) 0 mg_GO_·mL^−1^_chit. salt_ (sample without nanofiller); (**d**) 0.028 mg_GO_·mL^−1^_chit. salt_; (**e**) 0.055 mg_GO_·mL^−1^_chit. salt_; (**f**) 0.1 mg_GO_·mL^−1^_chit. salt_.

Figure 10 illustrates the degree of DNA damage in the BJ cells as the median of the percentage of DNA in the comet’s “tail” after the incubation of the cells with the hydrogels of the CH/LA/UMP system. The photos of comets constituting the cells’ response to the genotoxic effects of the above samples are shown in Figure 11. The results of the genotoxicity tests for variants of the CH/HCL/UMP system are shown analogously in Figure 12 and Figure 13.

The genotoxicity analysis performed for the hydrogels obtained from the chitosan lactate and chitosan chloride solutions with UMP showed that low doses of a nanofiller did not cause, according to the division introduced by Pereira et al. [31], DNA damage in the BJ cells. The median values of the percentage of DNA in the comet’s “tail” were obtained: 2.16% (the CH/LA/UMP system + 0.028 mg_GO_·mL^−1^_chit. salt_) and 2.95% (the CH/LA/UMP system + 0.055 mg_GO_·mL^−1^_chit. salt_) (Figure 10) and 3.41% (the CH/HCL/UMP + 0.028 mg_GO_·mL^−1^_chit. salt_) (Figure 12), which were not statistically significant compared to the negative control. The fact that the above variants of biomaterials do not exhibit genotoxicity features is proven by the microimages presented in Figure 11, photos (d) and (e), and in Figure 13, photo (d), which can be assigned to class 0, according to the classification given by Collins et al. [32].

The remaining samples were characterized by low genotoxicities: 8.26% (the CH/LA/UMP system + 0.1 mg_GO_·mL^−1^_chit. salt_) (Figure 10), 6.99% (the CH/HCL/UMP system + 0.055 mg_GO_·mL^−1^_chit. salt_), and 6.79% (the CH/HCL/UMP system + 0.1 mg_GO_·mL^−1^_chit. salt_) (Figure 12). The occurrence of statistical significance between the above types of chitosan systems and the negative control was found (*** *p* < 0.001). The recorded degree of DNA damage is also confirmed by the images collected in Figure 11, photo (f), and in Figure 13, photos (e) and (f), which can be classified as class 1 [32].

## 3. Materials and Methods

Chitosan from crab shells (CH) (Sigma-Aldrich, Poznan, Poland, product no. 50494, CAS no. 9012-76-4, weight of average molecular mass (M_w_) = 680 kg·mol^−1^), lactic acid (LA) (Sigma-Aldrich, Poznan, Poland, product no. L6661, CAS no. 50-21-5), hydrochloric acid (HCL) (Sigma-Aldrich, Poznan, Poland, product no. H1758, CAS no. 7647-01-0), graphene oxide (GO) (Sigma-Aldrich, Poznan, Poland, product no. 763713), and uridine 5′-monophosphate disodium salt (UMP) (Sigma-Aldrich, Poznan, Poland, product no. U6375, CAS no. 3387-36-8) were used in the preparation of the nanocomposite chitosan hydrogels.

The aqueous GO dispersion was obtained by sonicating GO flakes with the addition of ultrapure deionized water. The sonication process was carried out for approximately 1 h in an ultrasonic bath with a frequency of 40 kHz. The GO flakes had a linear size of 30–60 µm, and their edges were smooth and regular. 

### 3.1. Preparation of Nanocomposite Hydrogels

**Figure 14:**
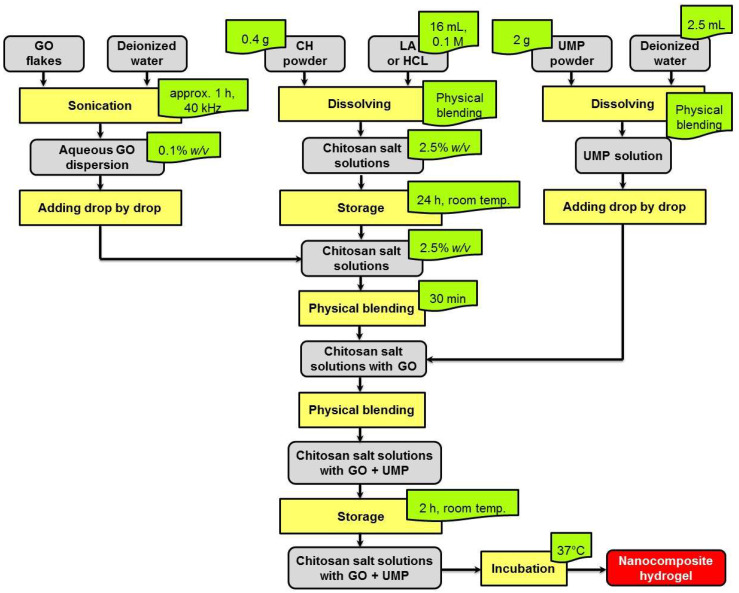
Scheme of preparation of nanocomposite chitosan hydrogels.

Figure 14 illustrates the steps involved in the preparation of the nanocomposite hydrogels.

In order to obtain the nanocomposite hydrogels, first, chitosan salt solutions (2.5% *w*/*v*) were prepared by dissolving 0.4 g of CH in 16 mL of 0.1 mol·L^−1^ LA or HCL. After thoroughly mixing, the samples were left at room temperature for 24 h. Then, a water dispersion of GO with a concentration of 0.1% *w*/*v* was added drop by drop into the solutions. Each sample was mixed for 30 min. The mass concentrations of GO in the chitosan salt solutions were 0.028; 0.055, and 0.1 mg_GO_·mL^−1^_chit. salt_. 

In the next step, solutions of the cross-linking agent (UMP) (2 g of UMP was dissolved in 2.5 mL of deionized water) were prepared. These solutions were then added gradually to the chitosan salt solutions with GO and were stirred at the same time. The samples were stored at room temperature for about 2 h. The prepared formulations were subsequently incubated at 37 °C to complete their gelation.

The chitosan salt solutions with GO (sols) were used for the rheological measurements. However, for the structural studies (FTIR spectroscopy) and surface analysis (SEM technique), the produced hydrogels were further processed by freezing at −20 °C and then lyophilizing under a pressure of 0.63 mbar and a temperature of −25 °C for about 48 h using the Martin Christ Freeze Dryer ALPHA 2-4.

As a control group, the unmodified chitosan hydrogels were prepared according to the procedure discussed in our article [27].

### 3.2. Rheological Analysis

Rheological tests were performed using the Anton Paar Physica MCR 301 rotational rheometer (Anton Paar, Warsaw, Poland) with a cone–plate measuring system (diameter—50 mm; angle—1°; truncation—0.048 mm). The sol–gel transition temperatures were determined based on oscillatory tests at a constant deformation value (angular frequency: ω = 5 s^−1^; strain amplitude: $\dot{\gamma }$ = 1%). The gelation processes were carried out under non-isothermal conditions, maintaining a constant heating rate of 1 °C·min^−1^. The solutions with GO were first cooled from room temperature (storage conditions) to 4 °C and then heated up to 60 °C.

### 3.3. Fourier Transform Infrared Spectroscopy

Fourier transform infrared (FTIR) spectra of the lyophilized hydrogels were obtained using a Nicolet™ iS50 FT-IR apparatus equipped with a monolithic diamond ATR crystal (Thermo Fisher Scientific Inc., Waltham, MA, USA). All spectra were recorded with 100 scans at a 4.0 cm^−1^ resolution in the wavenumber range of 4000–500 cm^−1^.

### 3.4. Scanning Electron Microscopy

The morphologies of the lyophilized chitosan hydrogels were determined using a Quanta 200 F SEM microscope (FEI, Hillsboro, OR, USA). The measurements were carried out in low-vacuum conditions (approximately 100 Pa) in a nitrogen atmosphere, and the accelerating voltage was 10 kV.

### 3.5. Biological Experiments

#### 3.5.1. Cell Culture

The analysis of the biological properties of the nanocomposite chitosan hydrogels was performed on the commercially available human fibroblast cell line BJ (ATCC^®^ CRL-2522™), purchased from the American Type Culture Collection (ATCC) (Manassas, VA, USA). The cells were cultured under standard conditions (5% CO_2_, 95% humidity, 37 °C) in Eagle’s Minimum Essential Medium (EMEM) (Sigma-Aldrich Corp., St. Louis, MO, USA) supplemented with 2 mM L-glutamine (GIBCO-BRL, Life Technologies Ltd., Paisley, UK), 10% *v*/*v* fetal bovine serum (FBS) (Sigma-Aldrich Corp., St. Louis, MO, USA), and 100 units/mL penicillin and 100 μg/mL streptomycin (both from GIBCO-BRL, Life Technologies Ltd., Paisley, UK). After exposure to accutase solution, the cells were passaged at 70–80% confluency.

#### 3.5.2. Preparation of Experimental Material for the Biological Studies

The experimental material (chitosan systems) was prepared under aseptic conditions in a laminar airflow cabinet (PCR Workstation by Labcaire Systems Ltd., Clevedon, UK).

#### 3.5.3. Assessment of Biocompatibility of the Modified Chitosan Hydrogels

The resazurin assay was used to evaluate the cytotoxic activity of the hydrogels. The assessment of the genotoxicity of the biomaterials was based on the alkaline version of the comet assay. The methods of carrying out the determinations were identical to the procedures presented in our articles [27,33].

#### 3.5.4. Statistical Analysis

Statistical analysis was performed in the statistical program SigmaPlot (Systat Software, Inc., San Jose, CA, USA). For each analysis, the normality test was performed using the Shapiro–Wilk test. In the case of the analysis of the cell viability level, a normal distribution was obtained; therefore, the statistical analysis between the two groups was performed using the Student’s *T*-test. For the comet assay analysis, no normal distribution was obtained; therefore, the statistical analysis between the two groups was performed using the Mann–Whitney test. Each of the analyses in the individual experiments was based on the results of three independent tests. In the graphs, the differences were statistically significant as follows: * *p* < 0.05; ** *p* < 0.01; *** *p* < 0.001 versus the negative control.

## 4. Conclusions

Based on the conducted research, it was found that the introduction of a nanofiller into the polymer matrix resulted in a decrease in the sol–gel transition temperature of the chitosan colloids while improving the mechanical properties of the systems. 

In turn, in the FTIR spectra of the hydrogels with GO, there were only changes in the intensity of the absorption signals recorded for the reference samples (unmodified hydrogels). Moreover, based on the SEM images, it can be concluded that the structure of chitosan systems enriched with GO is characterized by high porosity, similar to samples without nanofiller. There is connectivity between the scaffold pores, which can guarantee the effective colonization of the material and maintain the cell viability.

The biological studies have proven that hydrogels prepared from chitosan lactate solutions containing low concentrations of GO promote the survival of BJ cells at a level above 100%, but slightly higher compared to the viability of cells exposed to the unmodified CH/LA/UMP system, without inducing a genotoxic effect. The ability of BJ cells to proliferate in the presence of the above hydrogels makes the results of the biological research very promising, and the developed hydrogels are an attractive material for potential use as scaffolds in tissue engineering. However, more detailed, long-term studies are required to confirm the effectiveness and safety of these biomaterials. This is due to the fact that the use of graphene materials in medical sciences is associated with the risk of the permanent remains of these materials remaining in the body, the effects of which are not yet known.

## 5. Patents

Majsterek I., Modrzejewska Z., Pieklarz K., Tylman M.; Method for producing chitosan gels forming in the human body temperature, intended for injection scaffolds for breeding of nerve cells. Lodz University of Technology, Lodz. Poland. Patent application 235369. Publ. 29.06.2020 WUP.

Majsterek I., Modrzejewska Z., Pieklarz K., Tylman M.; Method for producing chitosan gels forming in the human body temperature, intended for injection scaffolds for breeding of nerve cells. Lodz University of Technology, Lodz. Poland. Patent application 243349. Publ. 07.08.2023 WUP.

## Figures and Tables

**Table 1:** The sol–gel transition temperature values of the chitosan solutions, without and with a nanofiller.

GO Concentration [mg_GO_·mL^−1^_chit. salt_]	Gelation Temperature (*T_gel_*) [°C]			
	CH/LA/UMP		CH/HCL/UMP	
0.00 (unmodified system)	6.4	37.3	10.0	41.2
0.028	13.1	22.7	16.5	25.3
0.1	*tan δ* < 1		*tan δ* < 1	

## Data Availability

The original contributions presented in the study are included in the article, further inquiries can be directed to the corresponding authors.

## Abbreviations

BJ	Human fibroblast cell line
CH	Chitosan
CH/HCL/UMP	System prepared from chitosan, hydrochloric acid, and uridine 5′-monophosphate disodium salt
CH/LA/UMP	System prepared from chitosan, lactic acid, and uridine 5′-monophosphate disodium salt
DNA	Deoxyribonucleic acid
EMEM	Eagle’s Minimum Essential Medium
FBS	Fetal bovine serum
FTIR	Fourier transform infrared spectroscopy
GO	Graphene oxide
*G*′	Storage modulus
*G*″	Loss modulus
HCL	Hydrochloric acid
LA	Lactic acid
SEM	Scanning electron microscopy
T	Temperature [°C]
*T_gel_*	Gelation temperature [°C]
UMP	Uridine 5′-monophosphate disodium salt
*tan δ*	Damping factor
ω	Angular frequency [s^−1^]
$\dot{\gamma }$	Strain amplitude [%]
